# Corrosion of Embedded Carbon Steels, Carbonation and Chloride Diffusion in Low-Clinker Hybrid and LC^3^-50 Cements: A Critical Review

**DOI:** 10.3390/ma19163521

**Published:** 2026-08-19

**Authors:** Asunción Bautista, Carlos Blanco, Francisco Velasco

**Affiliations:** Materials Science and Engineering Department, Universidad Carlos III de Madrid, Av. Universidad 30, 28911 Madrid, Spain; cbdiego@ing.uc3m.es (C.B.); fvelasco@ing.uc3m.es (F.V.)

**Keywords:** hybrid cement, LC^3^, steel corrosion, electrochemical monitoring, chlorides, carbonation

## Abstract

**Highlights:**

- The feasibility of low-clinker HC and LC^3^-50 for use in steel-reinforced structures is explored.
- A review of chloride diffusion, carbonation and corrosion monitoring is presented for HC and LC^3^-50.
- More extensively researched AAM and PC materials are used as references for discussion.
- Problems that carbonation or chloride diffusion tests designed for PC can have for evaluating other binders are examined.
- Problems with electrochemical measurement for slag-based or dense binders are discussed.

**Abstract:**

Portland cement (PC) is responsible for about 7–9% of the CO_2_ emissions worldwide. Alkali-activated materials (AAMs) are alternatives that have been intensely researched in recent decades, but some of their characteristics have hindered their extensive use in construction. Hybrid cements (HCs) and limestone calcined clay cements (LC^3^-50) are other, more innovative alternatives to PC, which seem easier to implement. Nowadays, there is active research about them, and pioneering studies about the durability of carbon steel reinforcements with these two types of binders are beginning to be published. In this review, the properties of HC and LC^3^-50 are briefly summarized and related with those of PC and AAMs. The uncertainties implied by using carbonation and chloride diffusion tests designed for PC to compare materials with different compositions are discussed. Using electrochemical methods seems logical to obtain full information about the durability of reinforcements in the innovative binders. However, these alternative binders have special features that can sometimes make the traditional electrochemical approach used yield misleading results. Factors such as the high resistivity some alternative mortars can exhibit, or the possible development of redox processes in the binder due to the nature of the precursors, must be borne in mind.

## 1. Corrosion of Steel in Concrete

Portland cement (PC) is the most extensively used material worldwide (if water is not considered) [1], although it exhibits poor mechanical properties for structures under tensile stresses. Modern load-bearing concrete structures use steel reinforcements to overcome the inability of concrete to handle those stresses. Nearly 100% of conventional bridges, highways, skyscrapers, and water-retaining facilities are built using steel-reinforced concrete. The durability of steel reinforcements embedded in PC concretes is guaranteed by the high alkalinity of the solution contained in the pores of the concrete [2], which gives rise to a stable passive layer on the surface of the embedded steel bars [3]. Besides highly soluble KOH and NaOH, this pore solution contains Ca(OH)_2_ [4]. This poorly soluble hydroxide is in equilibrium with the portlandite that has precipitated during the clinker hydration and that acts as alkaline reserve for the material.

Corrosion of the reinforcing bars in concrete can occur, however, when aggressive ions such as chlorides [5] penetrate through the concrete cover and reach the carbon steel bars, generating pits whose probability has been related to the [Cl^−^]/[OH^−^] ratio at the bar surface [2]. In cementitious materials, chloride ions primarily bind to C-S-H gel via physical adsorption, whereas calcium aluminate monosulfate (AFm) chemically reacts with chloride ions, forming Friedel’s salt [6]. The presence of free chloride ions significantly diminishes the service life of cementitious materials, while bound chloride ions are not corrosive as long as they remain immobilized in the binder [7]. Moreover, corrosion can also occur when the CO_2_ from the atmosphere diffuses, reacting with the hydroxides in the pore solution and decreasing the high pH that assures the passivity of the steel [8].

The environmental issues related to clinker production (which is more than 95% of the traditional PC composition) are well known. Clinker manufacturing entails the generation of greenhouse gases, air pollution (toxic emissions and dust), water consumption, and even land degradation (quarrying raw materials), and can also affect the health of the surrounding population [9]. About 7–9% of global CO_2_ emissions are related to the manufacture of traditional cements [10]. This is due to the fact that this compound is directly generated as a reaction product during the CaCO_3_ calcination thermal treatment to obtain CaO, and also the high temperatures (and high fuel consumption) that this process requires [11].

**Figure 1:**
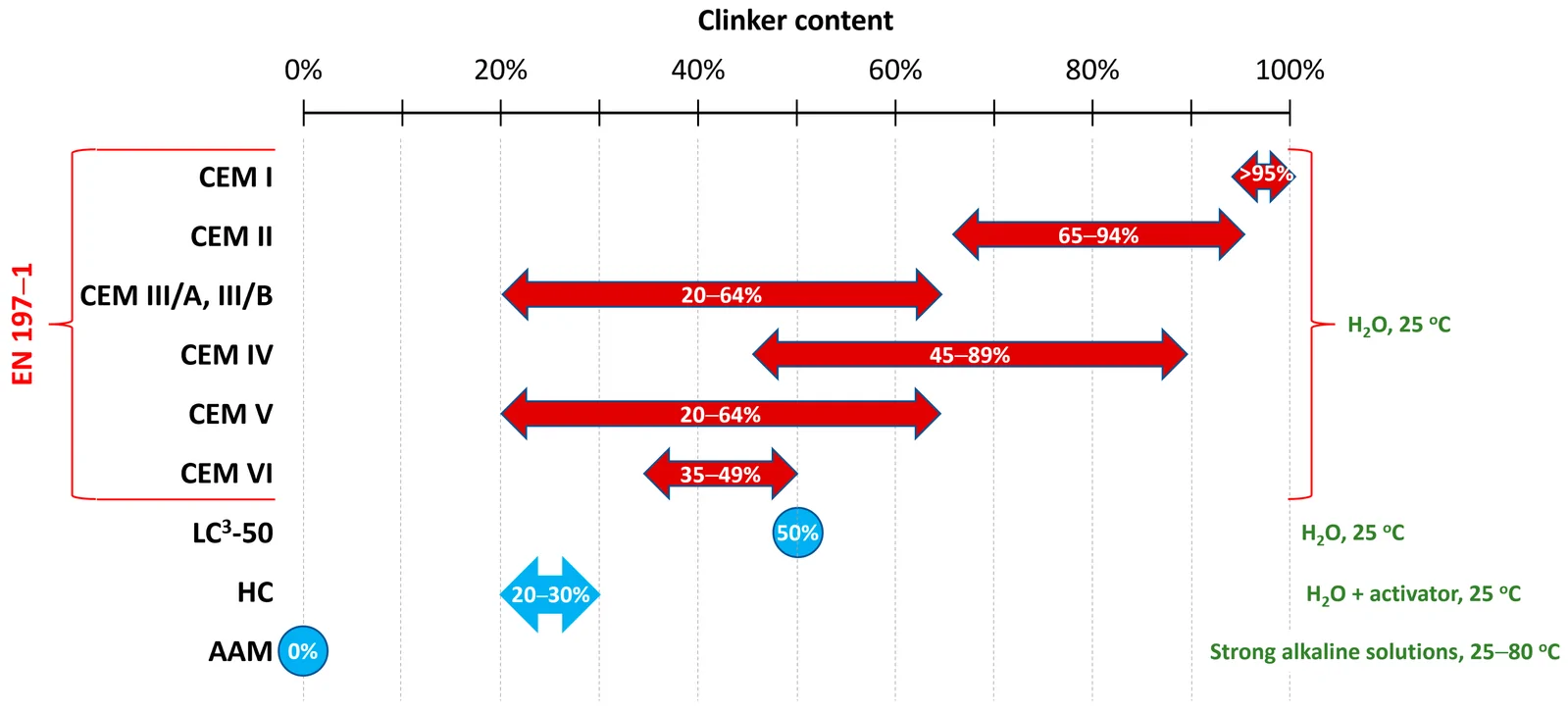
Content of clinker in standardized cements and alternative cementitious materials. Typical setting conditions and activators, which affect their implementability, are included.

In Figure 1, the standardized cement compositions can be seen, according to the EN197−1 standard [12]. Besides the clinker-rich PC (type CEM I), other cements with supplementary materials are standardized and are becoming more usual in the market. Nowadays, CEM II can be considered the most common, while other formulations with lower clinker content are often more difficult to find in the market and are usually employed for specific applications.

Moreover, new formulations such as alkali-activated materials (AAMs), which have no clinker content, or hybrid cements (HCs) and limestone calcined clay cements with 50% clinker (LC^3^-50), which have a reduced clinker content (Figure 1), are currently being explored as binder alternatives to PC. Bearing in mind the fact that most of the traditional cements are consumed for manufacturing reinforced structures, besides the mechanical, physical and microstructural characterization usually carried out for these new formulations, knowing how they interact with the embedded steel is a key point for maximizing the impact of their eventual implementation.

The possible use of alternative binders in reinforced concrete structures requires a deep characterization and a complete understanding of how the durability of the reinforcing bars can be affected. In the present review, relevant findings regarding the corrosion studies carried out in HC (Section 4) and LC^3^ (Section 5), as well as aspects that usually cause the loss of passivity of the steel (as carbonation or chloride diffusion), are considered. Published information regarding aspects such as porosity and pH, which can also affect the corrosion performance of embedded reinforcements, is included. Moreover, relevant aspects related to much more studied alternative binders (AAMs) are summarized in Section 3. Electrochemical techniques have been used for decades to obtain information about the corrosion performance of carbon steel bars in PC concretes and mortars [13], as they are able to give information in a non-destructive way and can be used not only in the laboratory, but also on a much larger scale [14]. However, recent studies have revealed that their direct use in alternative binders can be complex and prior reflection and/or research is necessary. A critical discussion of these aspects as well as of the problems that can imply the use of carbonation and chloride diffusion test is also included.

## 2. Review Methodology

In this work, articles published in journals relevant to the subject addressed were used as sources. The research sites used were Web of Science (all databases), ScienceDirect, Google Scholar and specific editorial sites. The paper mainly draws on peer-reviewed research papers, although conference proceedings and open-access theses were occasionally considered. The review focuses on studies dealing with characteristics of the binders that are directly related to the corrosion performance of the bars. The review primarily centers on the technical and engineering corrosion aspects, and other social, economic and political factors are not explored. Main keywords for the search carried included corrosion, passivation, chlorides, carbonation, hybrid cements, and LC^3^.

As the focus of the paper is on HC and LC^3^-50, the corrosion processes on PC and AAMs are not covered in detail and only specific points related to these binders that can help us to better understand the sections related to HC and LC^3^-50 are mentioned and referenced in the present article. Moreover, regarding limestone calcined clay cements, the present review is exclusively focused on the formulation with 50% of clinker, clearly the most studied one in the literature, though there are also other publications studying binders with calcined clay and different clinker contents, such as [15].

The study highlights points from existing literature regarding the limitations of using standardized tests—originally designed for conventional PC concrete—to evaluate alternative binders or compare binders of differing compositions. Furthermore, drawing on decades of experience conducting electrochemical measurements on steel embedded in reinforced mortar, the authors offer practical suggestions about the problems that can appear when strategies typically used with highly conductive liquid electrolytes are extrapolated to the measurement of steel in more resistive mortars or concretes.

## 3. Relevant Previous Experiences in Alkali-Activated Materials (AAMs)

### 3.1. AAM Composition and Pore Structure

In recent decades, major research endeavors have been devoted to AAMs as an environmentally friendly alternative to PC. AAMs are clinker-free binders obtained from the reaction between an aluminosilicate-rich precursor and a strong alkaline solution [16]. As precursor, solids considered wastes or byproducts of other industries are selected. Formulations based on blast furnace slag [17], fly ash [18], mixes of both products, metakaolin [19], agricultural waste [20], biomass ash [21] or volcanic ash [22] have been proposed, among others, in the literature. The composition of the precursor determines the structural gel formed: from Ca-poor precursors, such as fly ashes or metakaolin, N-A-S-H/K-A-S-H gels are formed, while C-(N)-A-S-H gels are generated from Ca-rich precursors, such as slag [16].

Activation is a clear weakness for AAMs. The activators typically considered are waterglass (sodium silicate in alkaline solution) or NaOH [23]. The contaminant manufacturing process of these activators [24] decreases their environmental benefits [25]; for this reason, different, more environmentally friendly alternative activators have also been explored [26]. The nature of the activator also affects the structure of the formed hydration products [27]. In any event, the alkaline activators are a dangerous solution because of their pH, which makes them not easy to use in construction because of safety requirements. Moreover, some precursors such as fly ashes are difficult to activate and require additional thermal activation at temperatures of about 60–80 °C [28].

### 3.2. Factors of AAM Binders Affecting Carbonation and Chloride Diffusion and Their Testing

**Figure 2:**
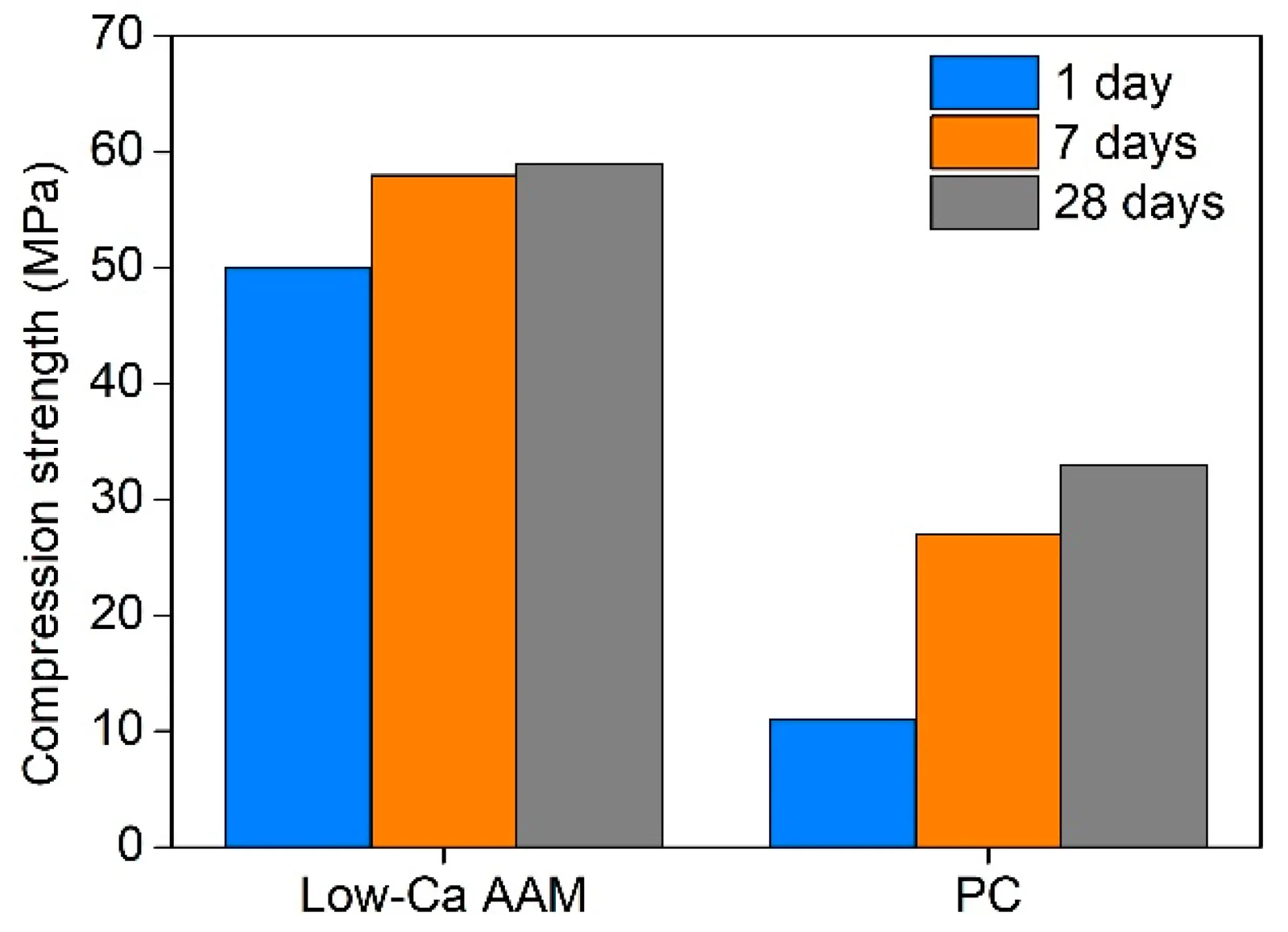
Compression strength of two concretes: a PC cured at 22 °C and a low-Ca alkali-activated material (AAM) (waterglass-activated fly ash) cured at 85 °C, for different setting times (based on [34,35]).

Recently designed AAM formulations have been shown to achieve low, refined porosity [29]. Such low porosities allow these binders to show good mechanical properties (Figure 2), sometimes much higher than that of PC [30]. Moreover, the refined porosities of many AAMs also favors their durability in comparison with PC when acid attack tests are carried out [31]. Nevertheless, it must be borne in mind that the chemical durability of AAMs can become a complex subject due to the fact that this term encompasses binders with very different chemical compositions and microstructures. For instance, low-Ca AAMs have been proven to react against sulfate attack with a mechanism different from that observed in PC or high-Ca AAMs [32], proving that low-Ca binders have a better sulfate resistance [33].

Regarding aspects directly related to the durability of carbon steel bars embedded in AAMs, such as corrosion due to chlorides and carbonation, it should be mentioned that the usual reduced porosity of these materials slows down chloride diffusion [36] and CO_2_ diffusion through the pores up to the reinforcements [37].

It has been reported for AAMs that carbonation tests carried out using high CO_2_ concentrations (>1%)—aimed at achieving a high acceleration of the process—can lead to misleading results [38]. For PC, CO_2_ concentrations ranging between 1% and 3% seem to cause changes and phase transformations similar to those identified for natural carbonation in the atmosphere, but at a higher rate [39]. On the other hand, when carbonation is carried out with 10% CO_2_, phases different from those formed in natural carbonation are found [40,41]. Unusually low pH values have been reported for fly ash AAMs exposed to 3% CO_2_ [42] due to the fact that, in this case, the activators affect pore chemistry and cause the reactions to be controlled by the equilibrium of Na salts [43]. The frequent use of 20% CO_2_ concentrations [44] can meaningfully affect the carbonation mechanism, and the problems could be even more marked for alternative binders than for the traditional ones.

A large part of published results reports that AAMs have better resistance to chloride diffusion than PC [45]. The chemical and physical properties of precursors, or the type and concentration of activators, are important for determining the chloride diffusion resistance of AAMs [45]. Chloroaluminates (Friedel’s salt) are not usually formed in AAMs, resulting in the chloride binding ability of AAMs being mainly determined by physical adsorption [45]. In any case, the finer pore structure of C-A-S-H gels enables high-calcium AAMs to present stronger chloride resistance than low-calcium AAMs [46].

Nevertheless, some published results regarding chloride diffusion must be reconsidered. The application of a positive polarization to the reinforcements can be seen as an easy, frequent way to try to accelerate Cl^−^ ingress through the mortar or concrete [47]. This strategy is considered in many traditional standards designed for Portland-reinforced concretes such as ASTM C1202 [48]. However, it must be borne in mind that not only Cl^−^, but also any negatively charged ions tend to move towards the positive pole, and transport charge can interfere in the results [49]. The pore solution of PC is already OH^−^-rich, but the AAM activated with alkaline solutions is much richer in this negative ion [50]. Hence, information regarding chloride diffusion obtained from current applications can be especially misleading when AAMs are considered [51]. The different compositions of pore solutions call into question the reliability of rapid tests that have been designed to evaluate chloride diffusion in PC-based concretes [45]. Moreover, the different chloride binding mechanisms in PC and AAM binders can also affect the results of accelerated chloride transport tests [52].

### 3.3. Other Factors of AAM Binders Affecting the Corrosion of Embedded Steel and Their Testing

As the compositions of pore solution in AAMs are different from that in PC, especially when high-Ca AAMs are considered, the passivation and depassivation mechanisms of the embedded steel can also be affected. The low-Ca AAMs have pore solutions whose composition [53] is relatively similar to that of PC [54], but more alkaline [55]. Higher chloride threshold values for depassivation have been suggested for AAMs (around 1.0–1.7 wt% for fly ash AAMs) than for PC (0.5 wt%) [56]. It has been suggested, from studies carried out in solution, that the traditional relationship of the pitting probability with the [Cl^−^]/[OH^−^] ratio [57], usually accepted for PC, should be modified for highly alkaline low-Ca AAMs to [Cl^−^]/[OH^−^]^3^ [55]. The corrosion monitoring with electrochemical techniques of steel in low-Ca AAMs seems to offer reliable results [49] and proves their effectiveness in passivating the steel reinforcement as fast and effectively as PC [58].

Another point that must be considered is the peculiar rheological properties of AAMs, which are very different from those of PC and can favor the generation of defects at the mortar–steel interface where corrosion nucleation easily occurs. This point has been verified in a systematic study carried out for low-Ca fly ash-reinforced mortars [59] and may have affected other published results that reflect poor corrosion protection [60].

**Figure 3:**
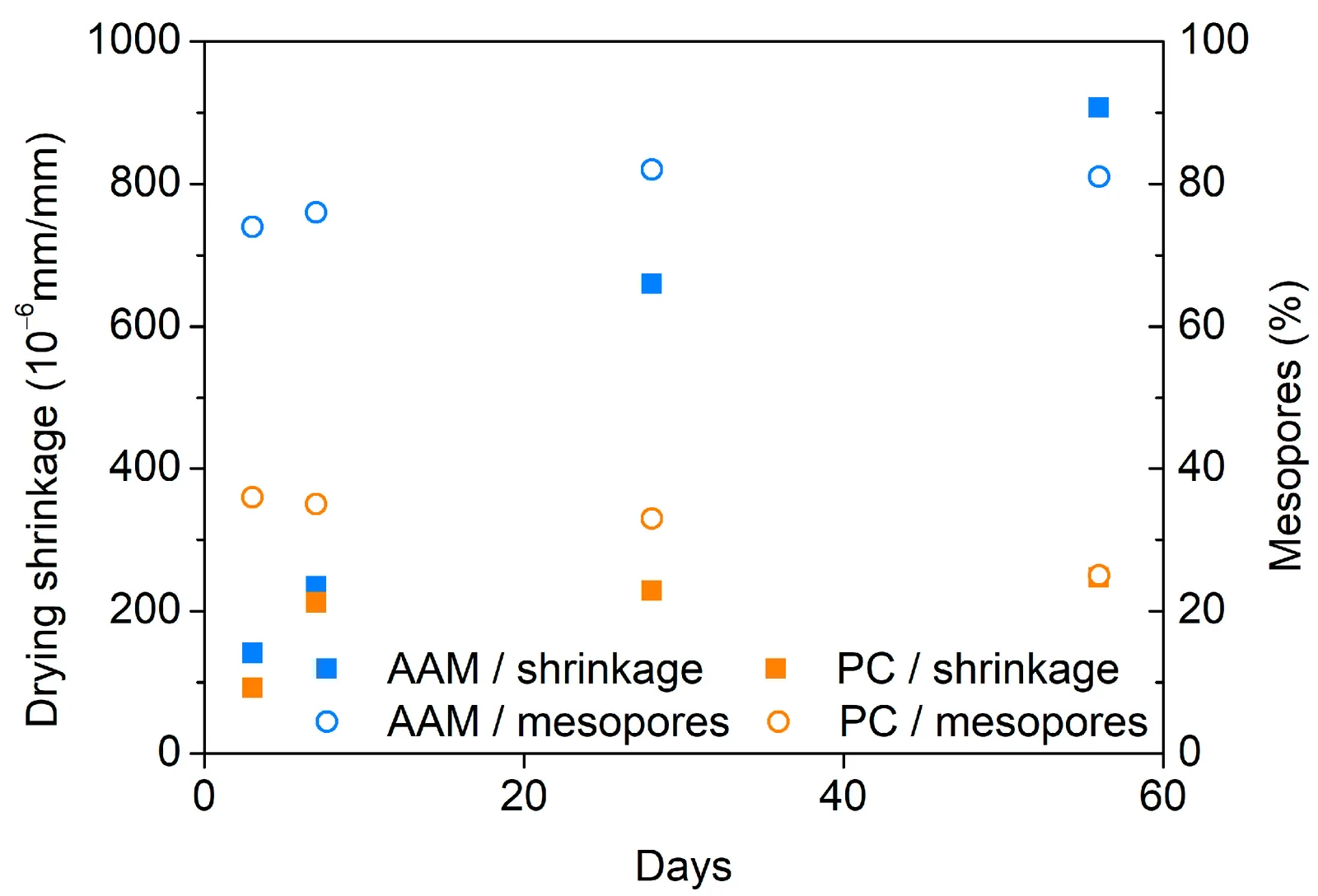
Example of the time evolution of the drying shrinkage and the amount of mesopores of a high-Ca AAM and a PC (based on [65]).

High-Ca AAMs also suffer much more marked shrinkage problems than PC [61], which have been related to their porosity, as refined porosity dramatically increases the stress in the pore walls during drying (Figure 3). Much higher drying stresses are generated in the mesopores than in the macropores [62]. These stresses can cause high deformations that the materials are unable to withstand [63], and microcracks or even cracks appear on the binders. Although intense activation can increase the mechanical properties by decreasing the pore volume in the binder, it also increases the shrinkage stresses [64].

Although cracks or microcracks often appear in high-Ca AAMs [66], the structural performance of AAMs is not usually compromised, as their porosity can be so reduced that the compression strength thoroughly exceeds that of non-cracked PC [63,67]. However, if reinforced structures are considered, the effect of the microcracking can become relevant for high-Ca AAMs. Properties that can meaningfully affect the durability of embedded steel bars such as chloride penetration [68] or even carbonation [66] or water captivity can be magnified if the binder is seriously damaged. The use of high concentrations of activators (NaOH or waterglass) increases the corrosion rate of embedded steel when the samples are exposed to chlorides [69], while increasing the mechanical properties and reducing the carbonation depth. This suggests that the reduced porosity increases mechanical properties and hinders the advance of the carbonation front, while also creating very localized cracks through which chlorides easily reach certain points of the steel surface. It has been verified that, unlike PC, chloride penetration does not correlate well with compression strength for these binders [70,71,72].

Moreover, as the refined porosity of AAMs poorly withstands drying processes, cracks can be generated or grown during the drying steps often involved in common laboratory corrosion tests [73,74]. These types of tests can seriously contribute to damaging the microstructure of these materials [68], affecting the results of the studies.

On the other hand, high-Ca AAMs are often manufactured from slag, exhibiting pore solutions with a high concentration of sulfur ions [75], which, when they are not fully oxidized to sulfate, give a green color to the mortar [76]. It has been reported that when electrochemical methods are used for monitoring the corrosion of steel bars embedded in slag-based AAMs, the redox process that these sulfur ions suffer in the presence of oxygen can mask the redox process corresponding to the corrosion of the bars [77]. The influence on open circuit potential (OCP) measurements of the redox process of the sulfur species has also been verified for AAMs where the precursors are a mix of slag and fly ash [78], decreasing the value corresponding to the steel.

In any case, AAMs have clear technical limitations that have hindered their use in construction. For high-Ca AAMs, the manufacturing process is more complex than that of PC, with setting processes that can be very fast and difficult to control with common set retarders [79], suffering large dimensional changes and very likely microcracking during curing and drying [63]. On the other hand, low-Ca AAMs, such as those based on fly ash, tend to show low early-age mechanical strength [80] or need a curing process that requires heating [81]. The mixes containing fly ashes with slag as precursors aimed at obtaining AAMs with more balanced properties can generate incompatible gels as hydration products, and give rise to binders with increased porosity and low mechanical strength [82]. All these limitations have limited the use of AAMs for practical applications and fostered the present studies in HC and LC^3^.

## 4. Studies in Hybrid Cements (HCs)

### 4.1. HC Composition and Pore Structure

HCs are innovative binders formulated essentially from 70–80% waste with pozzolanic activity such as slags, fly ashes or natural pozzolans [83,84]. As such, less than 30% clinker is present in their composition, which makes their carbon footprint meaningfully lower than that of PC, though higher than that of AAMs [85]. Moreover, the development of the hydrating process in these cements requires, as occurs for AAMs, the addition of an activator to achieve acceptable mechanical properties after curing [86]. This is the concept of HCs considered in this work, as it is the definition that was established for them in the initial proposal for these formulations, and that has been adopted in subsequent studies [87,88].

It is important to stress that this proposal seems able to overcome, with the use of this low amount of clinker and adding also small amounts of activators, the major rheological issues of the AAM [89]. Moreover, when slag is considered as a precursor, the addition of PC has proven to exert a positive effect on reducing the excessive drying shrinkage of AAMs [90,91], which can generate preferential diffusion paths for chlorides (Section 3). On the other hand, it has been reported that for HC manufactured from low-Ca precursors, such as fly ash, without resorting to additional thermal activation for curing [68], incomplete hydration can occur, giving rise to binders that can have their properties compromised in certain aspects, such as wear [92].

**Figure 4:**
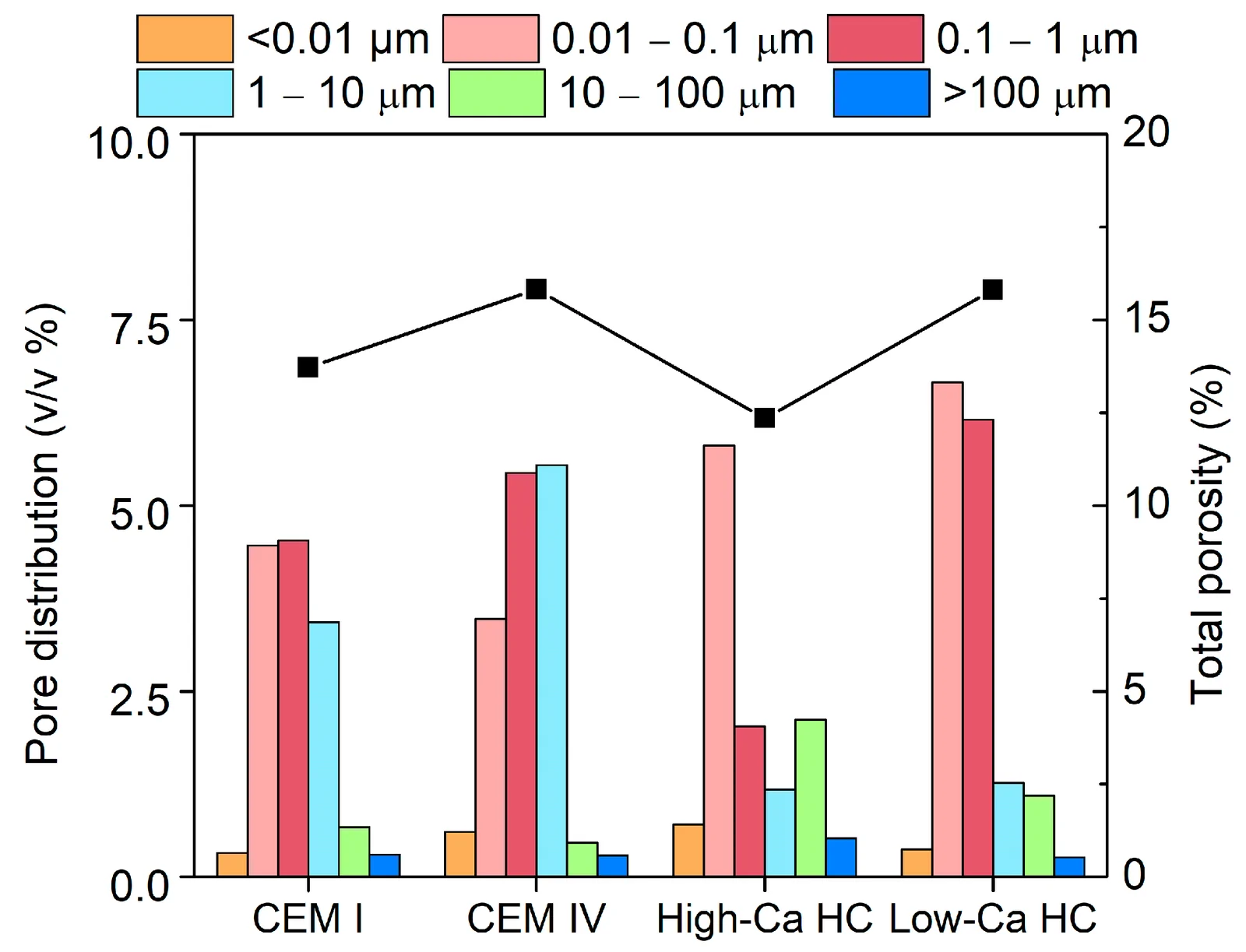
Pore distribution (columns) and total porosity (black line) of two PCs (types I and IV) and two HCs (based on [92]).

The hydration products of HC are a mix of the traditional C-S-H gel formed after the hydration of PC and the N-A-S-H/C-A-S-H formed in AAMs [93]. This last type of gels generates a pore structure that is much more refined than that of commercial cements (Figure 4) [94], filling voids and positively affecting the mechanical properties [95].

The heat generated by the hydration of the added PC accelerates the reaction of wastes without external heating [96,97], and the addition of an activator that increases the pH is necessary. Sodium hydroxide [98,99], waterglass [100,101,102] and mixes of both products [99,103] are considered as activators in previous studies, but the use of such high-alkaline solutions would clearly limit the manufacture of these binders outside the laboratory. If the activation is carried out with highly alkaline NaOH and waterglass solutions, the drying shrinkage of the HC is in between that of AAMs and PC [104].

Hence, the possibility of using solid, less-caustic salts as activators for achieving formulations that could be implementable in construction has been widely considered. Salts containing Na or K can be a very interesting alternative as activators, as they tend to form highly soluble hydroxides in the presence of the portlandite [97,105]. Portlandite first precipitates during PC hydration together with C-S-H gel. Later, the barely soluble Ca(OH)_2_ reacts with the Na or K salt to form soluble hydroxides and increase the pH.

Na_2_SO_4_ has been chosen as an alternative by many researchers, especially in recent publications [92,105,106,107,108,109], and its effectiveness seems to have already been proven. In the presence of sulfates, the formation of ettringite (an expansive phase) also occurs during the hydration of the precursors, resulting in a denser matrix [105]. However, for the hypothetical use of this binder in reinforced structures, it must be considered that the presence of this salt could favor the corrosion of the embedded steel [110]. If the sulfate amount is high enough, it cannot be discarded that the expansive formed could even damage the structure of the binder.

Carbonates are salts often considered as activators, both as Na_2_CO_3_ [94,111] and as K_2_CO_3_ [112], in spite of the well-known fact that carbonates cause expansive reactions in alkaline media [113]. These reactions can give rise to the formation and growth of crystalline products in a confined space [113] that can damage the binder matrix as microcracking can occur [94]. Even NaCl, which would be disposable for reinforcing structures because of its corrosivity, has been proposed [105]. Recently NaNO_3_ has been suggested as an activator that could be potentially suitable for reinforced structures [94].

The option of using solid salts as an activator for HC instead of highly alkaline liquids also has the advantage of favoring easier workability and better rheological control. Unlike AAMs manufactured from slag, whose problems of dimensional stability have been already mentioned [63], HCs manufactured from slag and activated with solid sulfates [114] or nitrates [94] are quite dimensionally stable during curing, and suffer shrinkages during drying that are somewhat lower that those determined for commercial PC.

### 4.2. Carbonation of HC Binders

For HC materials (20% PC + 80% slag), the progress of the carbonation front has been found to be faster than for clinker-rich PC under 1% CO_2_ at 65% relative humidity (RH) [86], but slower than for clinker-poorer CEM IV (Figure 1) under 3% CO_2_ at 75% RH [50]. These results can be easily related to the different amounts of precipitated Ca(OH)_2_ that exist. Portlandite in the pore solution reacts with the CO_2_ diffusing from the atmosphere, but as long as there is precipitated portlandite, this compound dissolves and keeps the pH of the pore solution at 12.6 [115]. When the alkaline reserve that the precipitated portlandite constitutes is consumed, the pH decreases and the carbonation front advances. The high-clinker PC has an alkaline reserve higher than that of cements with supplementary cementitious materials where part of the Ca(OH)_2_ is consumed in the pozzolan reaction [116]. However, the possibility that, depending on the specific HC formulation, the porosity controls the carbonation rate cannot be discarded. The carbonation process is usually controlled by the CO_2_ diffusion [117]. It has been reported that increasing the activator amount added to HC (that is to say, reducing its porosity) can decrease the progress of the carbonation front [118].

Under a mildly accelerated carbonation condition (only 1% CO_2_), concretes based on HC with 80% slag suffer meaningful loss of compressive strength when they are fully carbonated [86]. This result suggests that the low amount of portlandite precipitated in HC [119] is easily consumed and favors the CO_2_ reacting quickly with the structural gels that guarantee the structural properties of the binders [120]. The decalcification of the gels by carbonation has been proved to increase the matrix porosity [87], not only damaging the mechanical properties, but favoring the ingress of more aggressive agents.

### 4.3. Chloride Diffusion in HC Binders

On the other hand, another diffusing element that often affects the corrosion of the reinforcements is chloride. There are a limited number of studies on chloride diffusion in HC binders; some are in fact based on forced chloride migration [95,121,122], while other ones involve transport phenomena closer to those taking place in real structures [68,94]. It must be borne in mind that, as has been already discussed for AAMs in this article (Section 3), when currents are chosen to accelerate chloride transport, they can only be used to compare mortars or concretes with similar pore solution chemistry [123]. Hence, currents could offer misleading results if a HC is compared with a PC [121] or an AAM, or even another HC with a very different activator concentration. Results obtained up to now reveal that the penetration of the chlorides inside the mortar manufactured with HC is clearly related to their porosity, the presence of microstructural defects, and the phases formed. The refined porosity of HC favors the chloride binding on the pore walls by physisorption [94], limiting the amount of chlorides that penetrates through the cement and making it lower than in blended cements with supplementary additions or in high-clinker PCs [122]. However, the fact that the porosity of HC mortar is usually higher than that of AAM formulations with good mechanical properties makes the average chloride ingress in HCs higher than in AAMs [68]. In general, it can be assumed that the denser the structure of HCs, the higher the resistance to chloride penetration can be [95].

**Figure 5:**
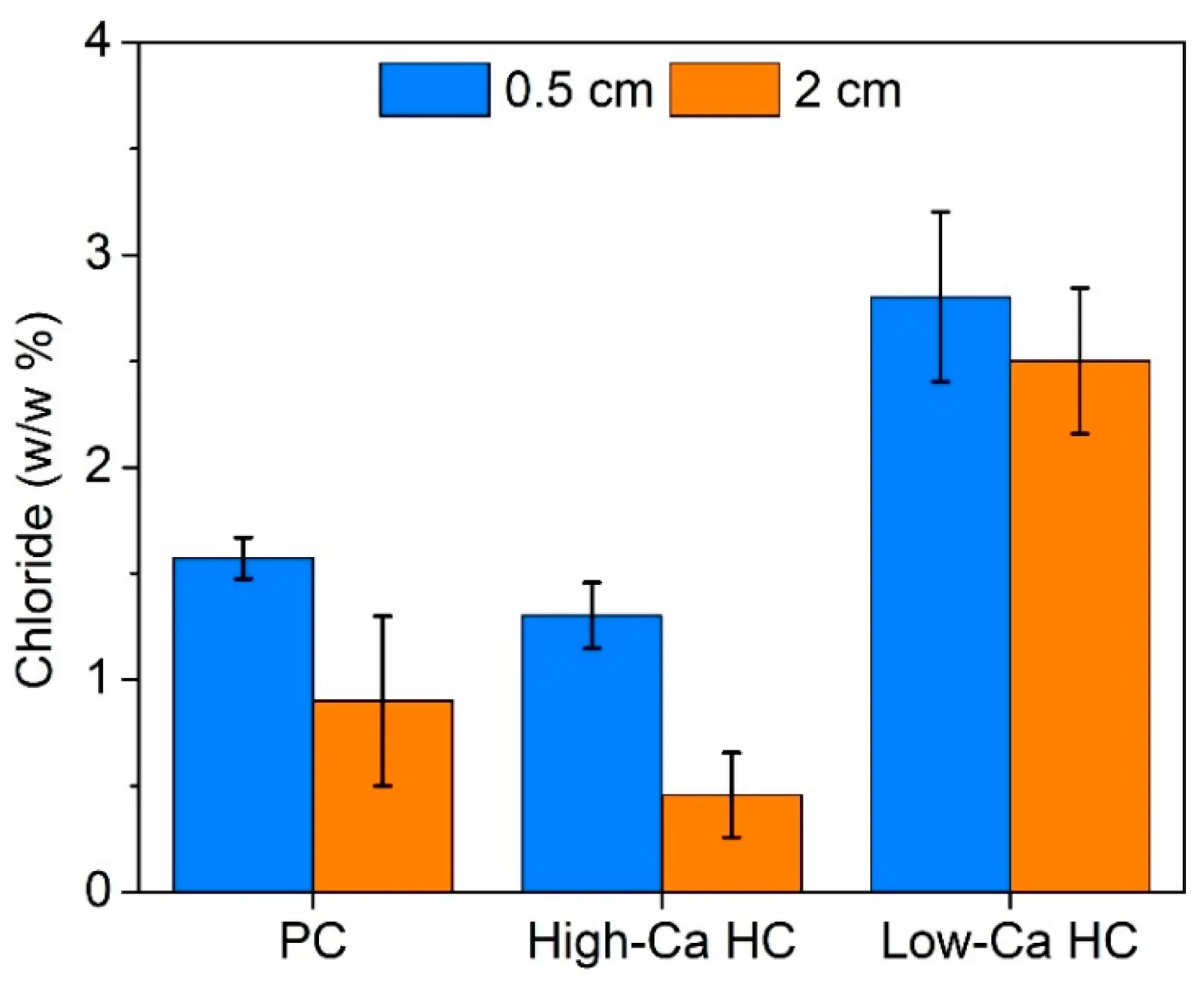
Amount of chlorides measured at two depths (0.5 and 2 cm) of three mortars: a PC, a high-Ca HC and a low-Ca HC. Test conditions can be found in [68].

In addition to the porosity volume and refinement, the literature published up to now suggests other factors that affect the chloride transport in HC, which are the nature of the precursors and the activators. The greater amounts of chloride that penetrate in HCs with low-Ca precursors compared to those with high-Ca in Figure 5 could be related to the difficulties in achieving complete curing after 28 days without thermal activation [68], even though chemical activators are used. Regarding the influence of the strong activation, high amounts of Na_2_SO_4_ lead to improved mechanical properties in the HC mortar, but can easily lead to slightly microcracked structures [68] through which chlorides can find preferential diffusion paths. These damaged microstructures can be overlooked if properties other than chloride diffusion are analyzed.

In a published study [94], activation with carbonates causes the highest chloride concentrations in the mortar after being submitted to the ASTM C1556-04 test (immersion without currents), in spite of being the lowest porous mortar considered in the study. Thus, the relevance microcracks can have for chloride transport is again confirmed. As previously indicated, the use of carbonates as activators can lead to expansive alkali–carbonate reactions [94]. When HCs have been activated with waterglass and the amount of PC exceeds 15% in the formulations, cracks have also been identified in the microstructure [95]. This indicates that the use of strong alkaline activators can also lead to damaged microstructures. Hence, the traditional compounds chosen as activators when the criterion is to reach high compression strength after curing cannot be the most adequate ones if the final goal is to manufacture reinforced structures using HC. As has already been demonstrated for AAMs (Section 3), very dense structures can give rise to high mechanical properties, but the binder structure is very prone to suffering cracking or microcracking.

Specifically for reinforced concrete structures, NaNO_3_ is being explored as an activator due to several interesting facts: (a) it does not lead to microcracked structures [94]; (b) it has been suggested as a possible corrosion inhibitor for steel in concrete [124]; and (c) it generates chloride-binding phases [94] (it forms AFm-NO_3_ hydrotalcite-type structures during the curing process [125,126], as in those identified for AAMs from MgO precursors, Section 3). After diffusion tests, lower chloride penetrations have been found in nitrate-activated high-Ca HC mortar in comparison with those activated with carbonates or those determined for PC with supplementary cementitious additions [94].

**Figure 6:**
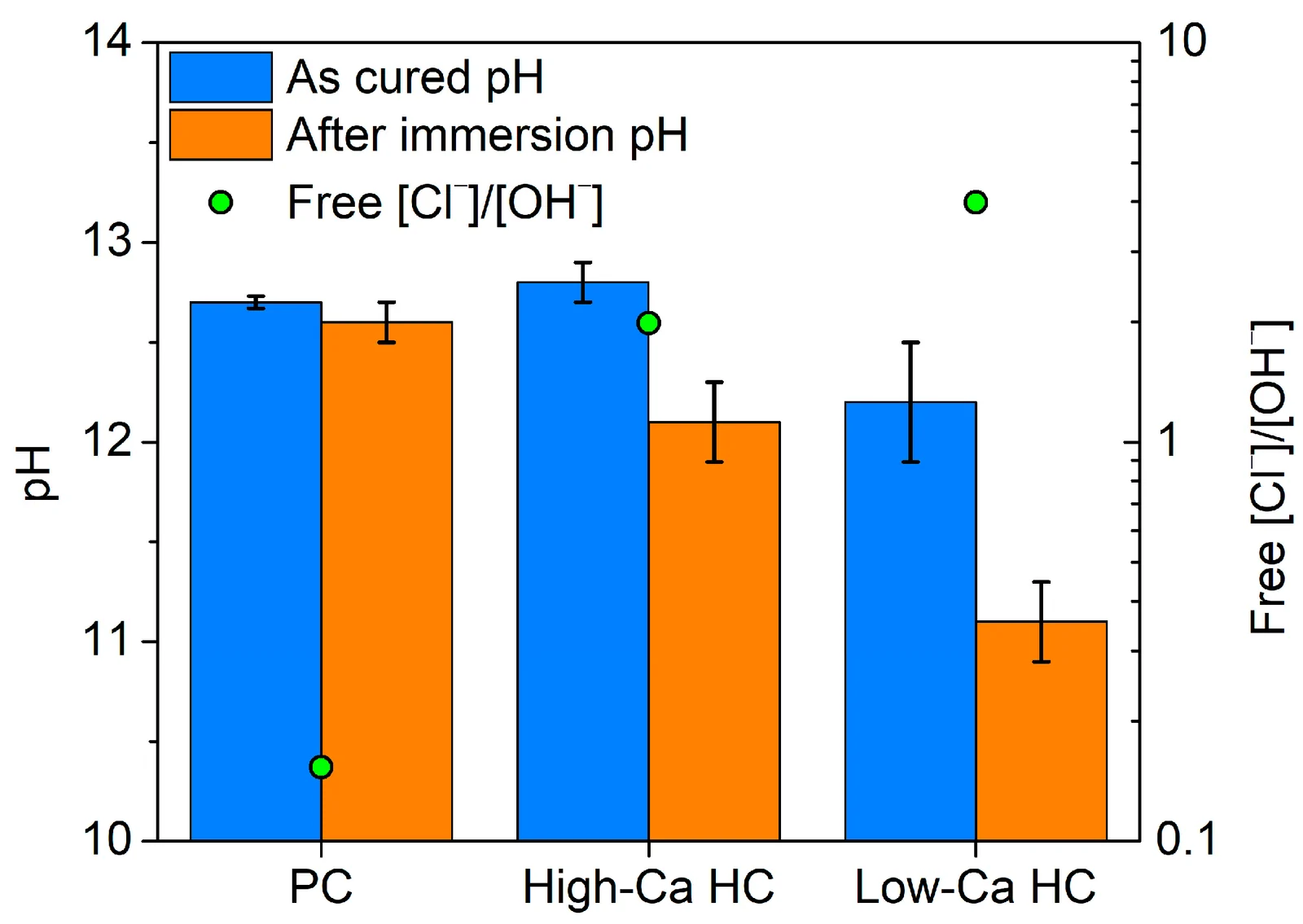
pH of three mortars measured at 0.5 cm depth from the surface, measured after 28-day curing and after 10 immersion/drying cycles plus continuous immersion for 74 weeks in 3.5% NaCl. The ratio between free chlorides and OH^−^ concentration at the end of the immersion is also included. More details in Ref. [131].

The pH of HC is another key point to determine the durability of the embedded carbon steel bars. The as-cured pHs determined for HC manufactured both from high-Ca or low-Ca precursors are alkaline enough to passivate the reinforcements (Figure 6). However, the in-service durability is highly dependent on the alkaline reserve of the binder, which determines not only the carbonation resistance, as has been noted earlier, but also the performance in environments with chlorides [127]. The onset of the pitting attack in the bars [128] and the rate it develops [129,130] are usually related to the [Cl^−^]/[OH^−^] ratio in the pore solutions, which is higher for HC than for PC (Figure 6). These results suggest lower critical chloride concentration for steel in HC. The pH also has a clear effect on the binding ability of the cementitious pastes [7], decreasing as the pH does. As the free chlorides in the pore solutions are the ones that directly foster corrosion [7], this is an important point. The low alkaline reserve of HC causes the pH to readily decrease after immersion cycles [131], making the ratio between the free Cl^−^ and OH^−^ in the pore solution higher and more dangerous (Figure 6).

### 4.4. Corrosion of Steel Embedded in HC

Published results regarding the direct evaluation of the corrosion performance of carbon steel bars in concretes or mortars manufactured with HC are still very limited. A summary of relevant publications found can be seen in Table 1. Most of this research uses d.c. electrochemical measurements to obtain information about the corrosion rate, but the morphology of the attack has also been characterized [68,131] and the usefulness of a.c. electrochemical impedance spectroscopy (EIS) has been explored [68,131]. 

**Figure 7:**
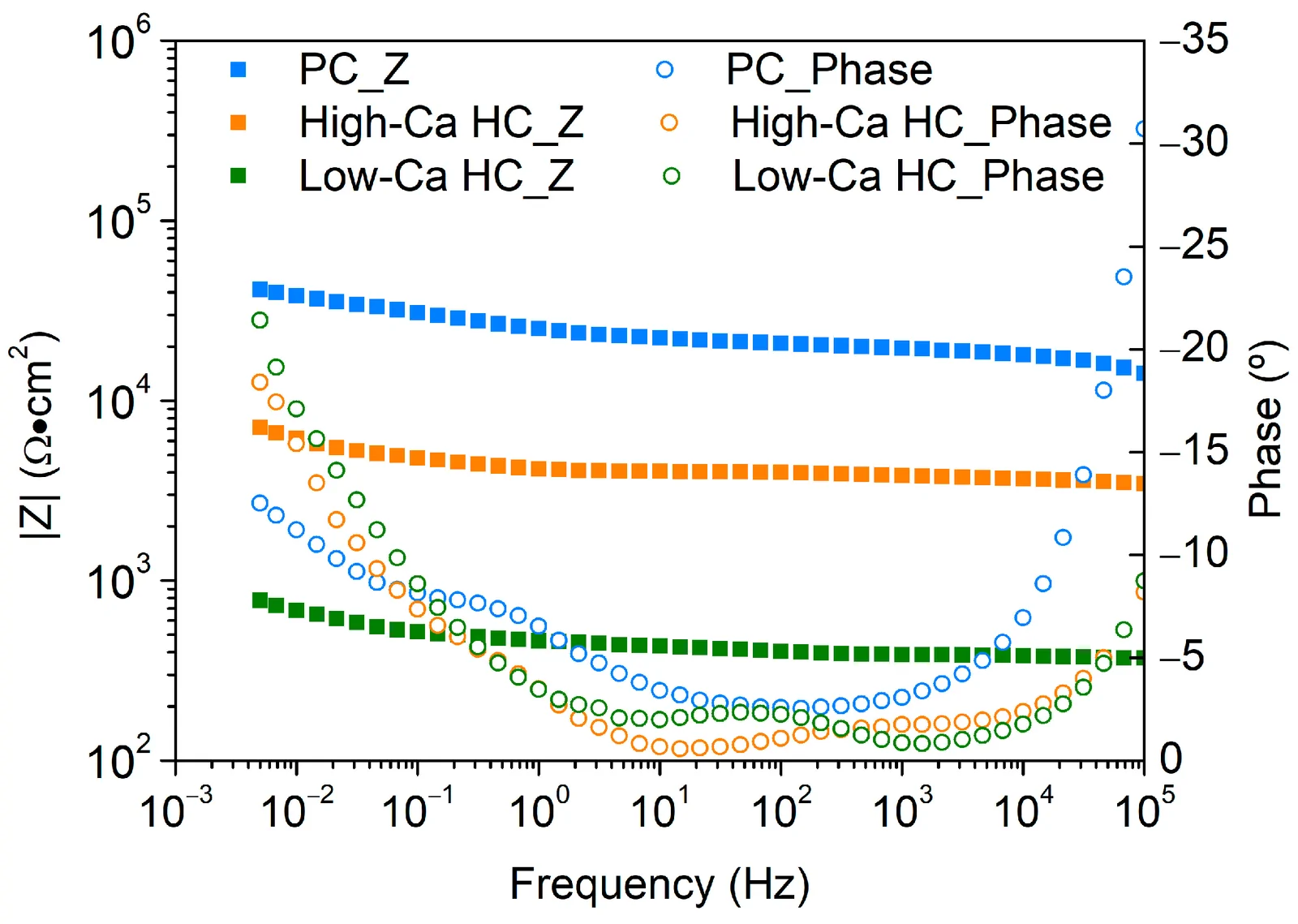
EIS spectra of the steel–mortar systems (in Figure 5) measured after 10 immersion/drying cycles plus continuous immersion for 37 weeks in 3.5% NaCl solution. More details in Ref. [131].

EIS studies carried out in different mortar types and tested in corrosive environments for steel have been shown to be unable to offer accurate information about the charge transfer resistance (R_t_) that determines the corrosion rate of the steel bars. The spectra in [68,131] are acquired up to 5 mHz without low-frequency noisy interferences. As can be seen in the spectra in Figure 7, at the lowest frequencies, no clear information about the R_t_ of the reinforcement can begin to be ascertained as most of the spectra corresponds to the mortar resistance. Acquiring a few more low-frequency points (it can be technically done without meaningful noise up to 2.5–1.0 mHz in these systems) [132,133] probably could not solve this relevant uncertainty. The lack of information about corrosion kinetics in the EIS spectra can appear for two, often related, reasons: first, because the mortar resistivity is too high; second, because the R_t_ has values lower than or very similar to the mortar resistance. An attempt to obtain semiquantitative information from spectra was carried out in [68,131], relating the corrosion rate with the inverse of the difference between the impedance corresponding to lowest frequency measured with reliability and the mortar resistivity.

**Table 1:** Summary of relevant publications about the corrosion of steel reinforcements embedded in HC.

Article	Material	Precursor	Activator	Corrosive Condition	Corrosion Tests Carried Out	Conclusions About Steel in HC
[100]	Mortar w/b: 0.5	30% PC + 70% Fly ash	15% waterglass, 85% 12.5 M NaOH solution (liquid)	Addition of chlorides during manufacturing 95% RH for 94 days + 30% RH for 180 days + 95% RH for 205 days	OCP Polarization curves Galvanostatic pulses	Passivation at 0.4% Cl^−^ (similar to PC) Lower corrosion rate when activated with waterglass than in other HC, PC and AAM with 2% Cl^−^
	Mortar w/b: 0.4		8% Na_2_Si_2_O_5_ + 8% Na_2_CO_3_ (solid)			
[86]	Concrete w/b: 0.45	20% PC + 80% slag	Na_2_SiO_4_ 44.26 kg/m^3^, NaOH 12.19 kg/m^3^ (liquid)	Carbonation at 1% CO_2_, 65% RH for 99 days	OCP Linear polarization Visual evaluation	Corrosion rates correspond to active corrosion from the beginning of the exposure
[134]	Concrete w/b: 0.48	20% PC + 80% fly ash	NaOH 54.79 kg/m^3^, Na_2_SiO_4_ 220.75 kg/m^3^ (liquid)	Carbonation at 1% CO_2_, 65% RH for 150 days	OCP Linear polarization Polarization curves Visual evaluation	Lower corrosion resistance than in carbonated PC
[135]	Concrete w/b: 0.58	20%PC + 80% fly ash	NaOH 0.23 kg/kg of binder, Na_2_SiO_4_ 0.23 kg/kg of binder (liquid)	Wet–dry cycles in 3% NaCl for 245 days	OCP Linear polarization Cross-section losses	Corrosion resistance and section losses close to that in PC
				Impressed voltage steps in 3% NaCl for 59 days		
[101]	Concrete w/b: 0.48	20% PC + 80% fly ash	Waterglass 268.1 kg/m^3^ (liquid)	Immersion in 3.5% NaCl for 360 days	Polarization curves Linear polarization	50% lower i_corr_ than in AAM from slag + fly ash after 360 days
[68]	Mortar w/b: 0.42	20% PC + 80% slag	5% Na_2_SO_4_ (solid)	10 wet–dry cycles (partial immersion in 3M NaCl + drying at 45% RH)	OCP EIS Weight losses Pit morphology Visual evaluation	Estimated corrosion higher than in AAM and commercial cement Less localized corrosion morphology than PC
	Mortar w/b: 0.37	20% PC + 80% fly ash	3% Na_2_SO_4_ (solid)			
[131]	Mortar w/b: 0.42	20% PC + 80% slag	5% Na_2_SO_4_ (solid)	10 wet–dry cycles (partial immersion in 3M NaCl + drying at 45% RH) + 74 weeks continuous immersion	OCP EIS Pit morphology Visual evaluation	In HC, the pits appear on the most-stressed regions of the bars In HC from fly ash the highest corrosion rates are identified
	Mortar w/b: 0.37	20% PC + 80% fly ash	3% Na_2_SO_4_ (solid)			

The ohmic drop through the mortar can also meaningfully distort the values obtained using d.c. techniques. If this parameter is not properly compensated, ohmic drops of the magnitude close to that of the mortar/concrete resistances can be easily obtained. The mortar resistivity has been compensated for electrochemical d.c. measurements in previous papers on steel in mortar from commercial cements [132,133], but also in a pioneering publication with HC formulations [100]. However, no information regarding implementation of ohmic drop compensation appears in other, more recent studies [86,101,134,135].

If proper ohmic drop compensation could be successfully implemented in HC systems, the quantification of the corrosion rates of steels embedded in low-Ca HC by applying d.c. techniques could be reliable. Results of measurements performed in steel in fly ash HC mortars confirm the coherence between the results of lineal polarization measurements and section losses [135], probably due to the high electrical conductivity of the binder under study.

**Figure 8:**
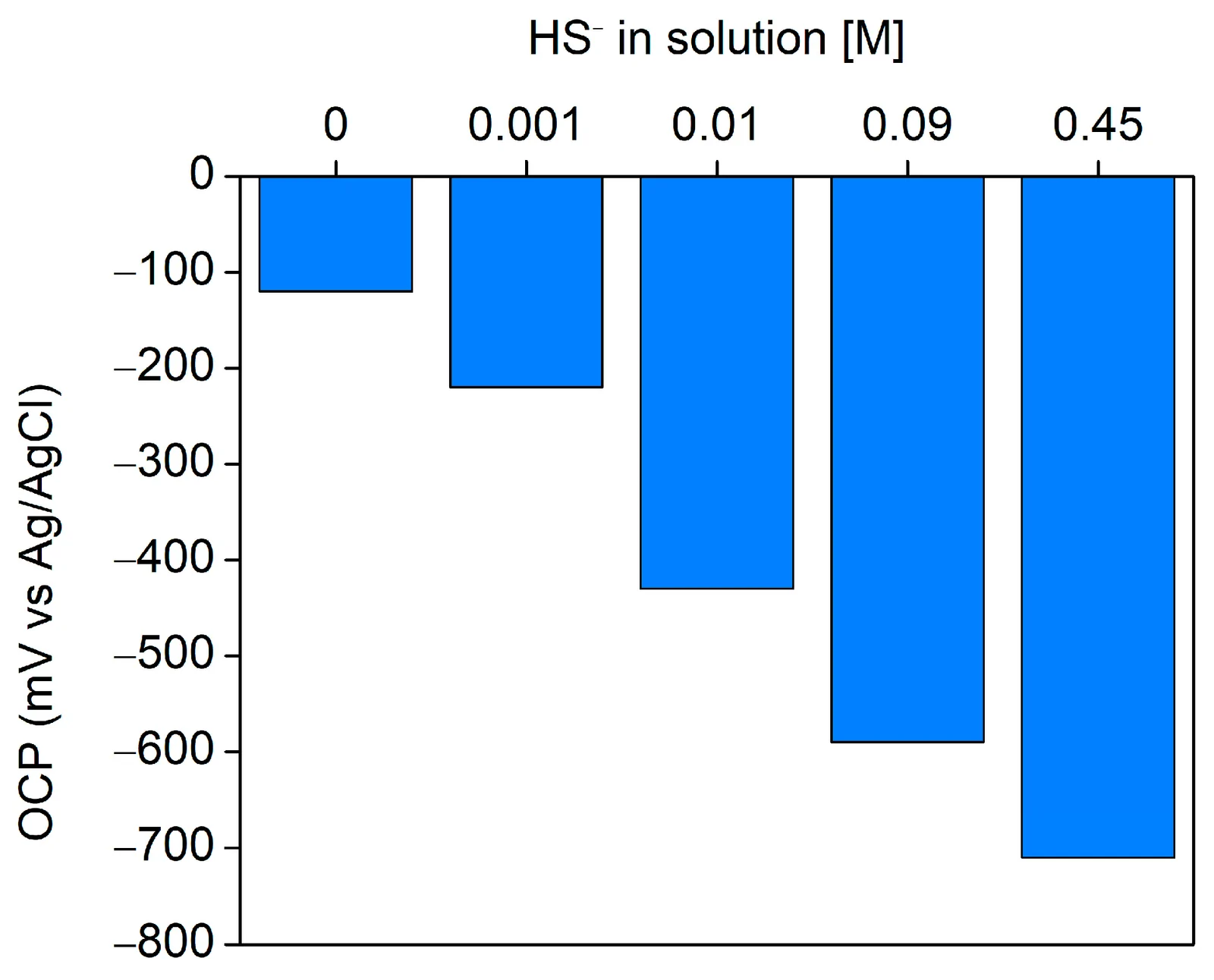
Open circuit potential measured on steel, after being immersed for 12 days in a 0.80 M NaOH solution with different HS^−^ concentrations (based on data of [77]).

On the other hand, for systems manufactured with sulfide-rich precursors such as slag, the interference on the OCP measurements and corrosion rate determination already discussed for high-Ca AAMs must be borne in mind. In Figure 8, it can be seen that the OCP of steel in alkaline solutions with increasing amount of sulfides decreases in value in spite of the absence of chloride or other corrosive agents in the media. This confirms that apparently active potentials and the measured current densities must be due to oxidation of the sulfur species present in the pore solution. This fact has not been considered in several previous studies of steel in HC mortars from slag [68,86,101,131], but has been used in a recent study to justify apparent experimental incoherencies discovered during the investigation [134].

**Figure 9:**
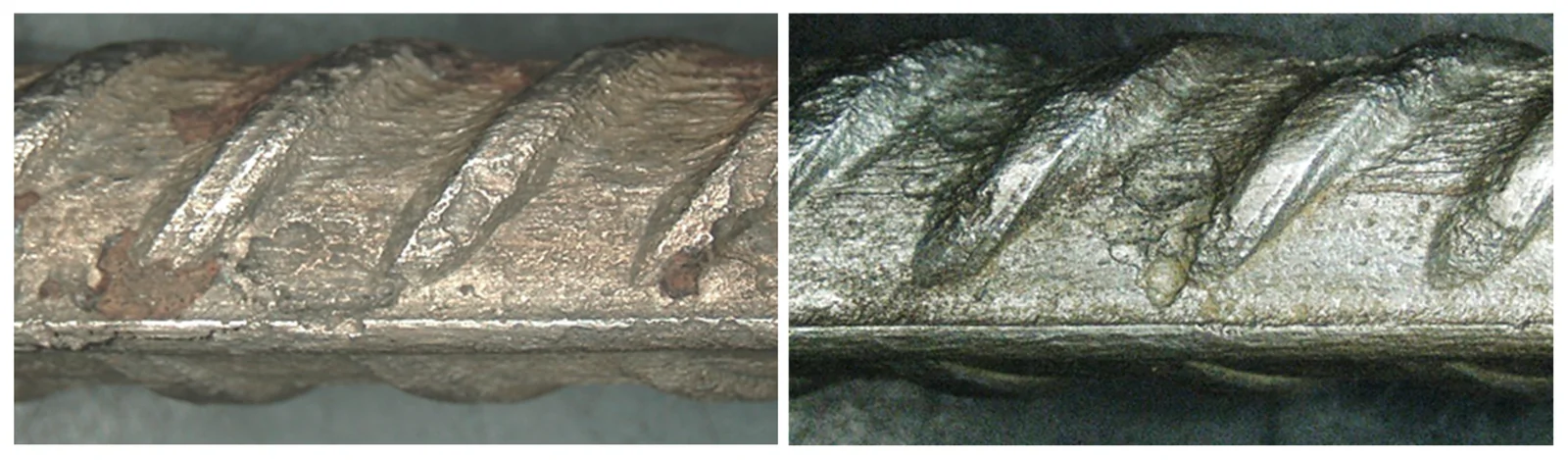
Pits formed in steel reinforcements embedded in HC mortars (**left**, low-Ca, **right**, high-Ca precursors) after 2.5 years of immersion of the reinforced system in saline solution [136].

In spite of the marked masking effect that the sulfurs exert in the electrochemical measurements of the reinforcements for steel in high-Ca HCs, visual observations and optoelectronic studies inform that, in the cyclic exposure conditions detailed in [68,131], chlorides favor the pitting attack of the bars (Figure 9), although with lower intensity than in the HC from a low-Ca precursor exposed to the same conditions.

As Table 1 shows, it is difficult to find full consistencies among the results. In addition to previously discussed monitoring issues, as Table 1 states, precursors, activators and their quantity, water-to-binder ratios and exposure conditions differ considerably, meaning that corrosion results can easily be different.

Hence, HC can be a more implementable alternative to AAMs, especially when solid salts are used as activators as they have much fewer technical issues—related to a lower hazard level of the activator, higher dimensional stability [137], better workability [87] and longer setting times [138]—for use in construction. However, more durability studies are necessary to reach this objective with reliability. At the same time, the influence of the activators on the electrochemical durability of embedded reinforcing bars still presents uncertainties that must be solved.

## 5. Studies in Limestone Calcined Clay Cement with 50% Clinker (LC^3^-50)

### 5.1. LC^3^-50 Composition and Pore Structure

It has been reported that the supply of commonly used supplementary cementitious materials (fly ash and blast furnace slag) will be reduced in the coming years due to the retirement of coal-fired power plants and the retirement and limited lifetime of blast furnaces [139]. Hence, limestone calcined clay cements with 50% clinker (LC^3^-50), which do not use this type of addition to reduce the clinker content, have emerged as promising low-clinker binders. Introducing calcined clays into LC^3^, together with limestone, reduces the clinker content by up to 50% in an easily implementable binder that only requires neutral water for setting [140]. Moreover, in its composition, LC^3^-50 has 30% calcined clay, 15% limestone and 5% gypsum [141]. The wide availability of clays [142], as well as their pozzolanic behavior after being activated, results in an environmentally friendly alternative to PC.

The structure of the kaolinite present in the clay, a 1:1 layer combining tetrahedral silicate sheets with octahedral aluminum oxide sheets [143], collapses under the effect of calcination [144], turning kaolinite into metakaolin, with pozzolanic properties. The reaction of metakaolin with Ca(OH)_2_ generates C-(A)-S-H gel, which refines the porosity of the binder [145]. The initial kaolinitic content of the clays is an important factor regarding the kinetics of the reaction, as well as the degree of pore connectivity refinement achieved. Increasing kaolinite values up to 65% in the clays results in faster kinetics and lower porosity [146]. Above that value, the porosity does not get refined further, as the critical pore entry radius has already been reached [146]. This coincides with mechanical properties: increasing the amount of kaolinite above 60% in clays provides little (or no) benefit in compression strength [147]. The clinker content also influences reaction kinetics, as portlandite is needed to sustain the pozzolanic reaction with metakaolin [148]. In any case, it is important to highlight that research on different activation methods (such as mechanical activation [149,150]), about the use of low-grade kaolinitic clays [151], or even joining both lines (e.g., mechanical activation of a muscovite rich-clay [152]) is taking place.

The microstructure formed during hydration, originating from the release of alumina found in the clays and the CaCO_3_ present in the limestone, results in the formation of C-(A)-S-H and carboaluminate phases, while gypsum enables stabilization of ettringite [141,146,153,154].

A weakness that can be found for LC^3^-50 cements is their increased water demand, as one of the most significant characteristics of clays is their ability to adsorb water [155]. This creates the necessity to introduce water-reducing agents or superplasticizers in order to avoid increasing the amount of water required to obtain the required workability values [156]. These compounds act by dispersing the cement particles in the water, allowing them to be hydrated. As low quantities of these agents are used in the formulation of LC^3^-50, the benefits they provide surpass the negative environmental impact of superplasticizers [157].

A property that dictates the behavior of these binders when exposed to corrosive environments is their refined porosity. Due to the nature of the network, it yields good mechanical properties, comparable with other environmentally friendly options, such as fly ash-based HC [158]. However, it must be borne in mind that the pozzolanic reaction consumes portlandite, which reduces the alkaline reserve of the binder [153,159,160,161].

LC^3^-50 systems tend to show higher autogenous shrinkage values compared with PC, due to their faster hydration [162]. Nevertheless, drying shrinkage is reduced due to the nature of the pore network [163], resulting in similar total shrinkage values after prolonged exposure [162,163]. No reference about cracked microstructures due to stresses during curing and drying has been found by the authors for LC^3^-50.

### 5.2. Carbonation of LC^3^-50

The effect of carbonation in these binders has been reported in the literature for the interval of 1–20% CO_2_ [160,161,164,165,166]. The reduced amount of portlandite present, as a consequence of the low-clinker formulation, lowers the alkaline buffer available to maintain the pH of the pore solution, as occurs in HC (Section 4). Moreover, coarsening and increased interconnectivity of the pore network [160,164] have been detected during carbonation of LC^3^-50 with 3% CO_2_. This poorer carbonation resistance of LC^3^-50 (vs. PC) has been identified for mortars carbonated with 1% [165], 3% [167], 5% [165] and 20% CO_2_ [166]. During the carbonation (with 20% CO_2_), the amount of 1–3 nm pores usually increases due to calcification, while the amount of larger pores (>10 nm) is usually reduced due to CaCO_3_ precipitation [166]. Carbonation tests of LC^3^-50 carried out for 14 days at 20% CO_2_ and 65% RH identified C-(A)-S-H and ettringite decalcification by XPS [145].

When accelerated carbonation tests are carried out, the progress of the carbonation front is controlled by water condensed in the pores (the plugging effect) [165]. The plugging effect does not appear under natural carbonation conditions, although it is detectable at 1% CO_2_ test and becomes more evident as the CO_2_ concentration increases [165]. The small pores condense higher water amounts than pores of a bigger size, so LC^3^-50 binders, with a more refined pore structure, are more sensitive to this interference than the PC, whose porosity is coarser [168]. These results are coherent with the conclusion reached by other authors studying AAMs regarding the fact that accelerated tests resorting to high CO_2_ concentrations especially affect the carbonation mechanism for binders with very-refined porosity [42]. On the other hand, the carbonation products formed in LC^3^-50 tested at 3% CO_2_ have been considered similar to those formed under natural exposure [160].

Under natural carbonation conditions, LC^3^-50 pastes have proven to have poorer carbonation resistance than PC pastes [169], although the paste porosity is quite different from that of mortars and concretes. In fact, a simulation study shows the differences in natural carbonation among eight cities of the world for the same LC^3^-50 concrete, indicating that the formulation of the LC^3^-50 (e.g., water-to-binder ratio) should be changed to not exceed a limit depth in the next fifty years [170].

After tests carried out in highly aggressive conditions (20% CO_2_, 80% RH, 30 °C, 30 days), the mortar samples were fully carbonated, as the phenolphthalein test informs [166]. Changes in the mechanical properties in mortar samples of 2.5 cm Ø and 2.5 height due to carbonation were barely noticeable, assuming that the positive effect of CaCO_3_ filling the pores balances the decarbonation suffered by the structural gels [166].

### 5.3. Chloride Diffusion in LC^3^-50

Considering corrosion aspects, the precipitation of carboaluminate phases allows for lower ionic transport and reduces permeability [171]. Moreover, the tortuosity of the pore network [172], the low porosity and smaller pore sizes [173] result in an increased resistance to chloride diffusivity in LC^3^-50 [171]. The advantages of using LC^3^-50 for reducing chloride concentration profiles have been verified [174]; both penetration depth and concentration of chlorides are smaller in LC^3^-50 due to the lower permeability that can be achieved [167].

**Figure 10:**
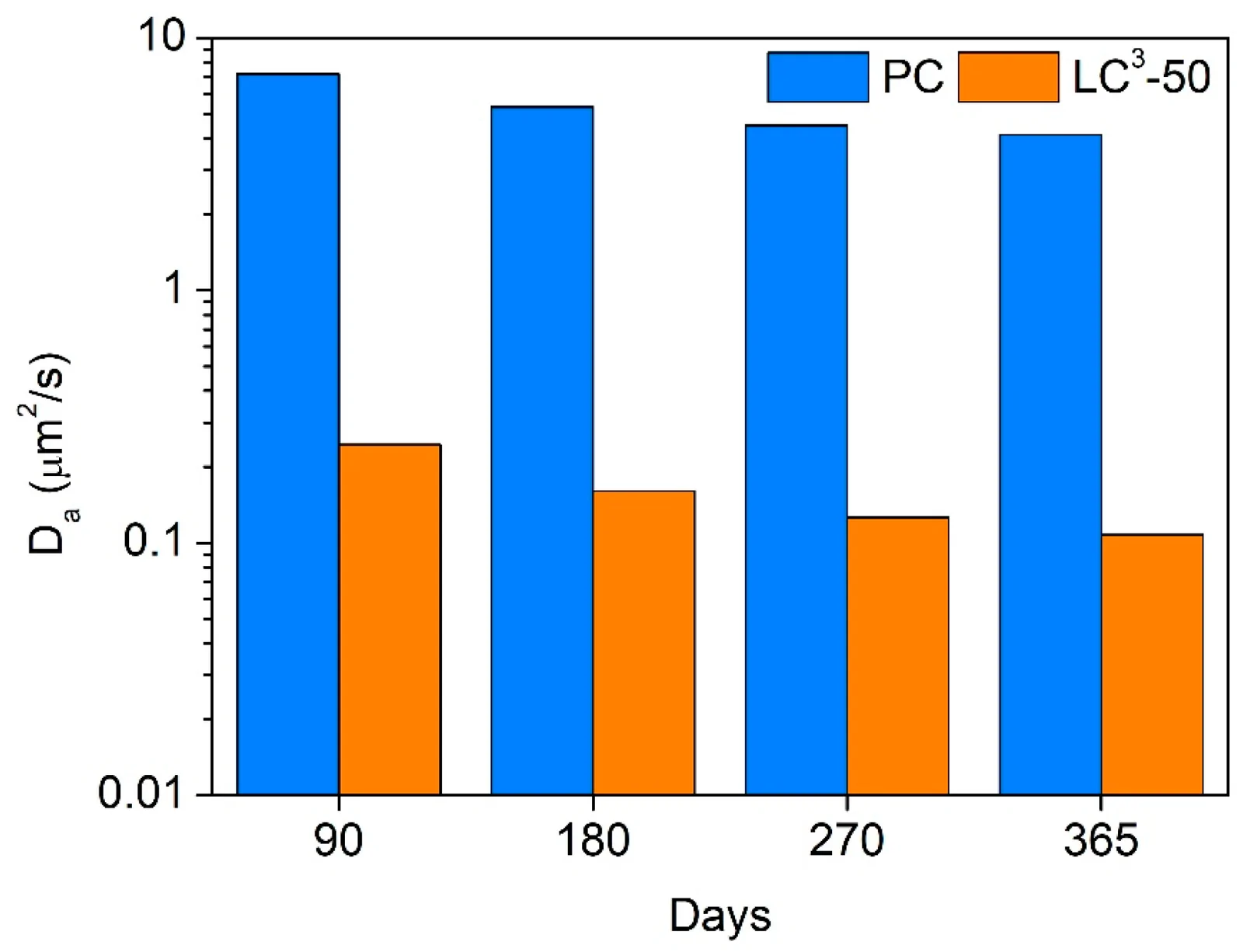
Apparent chloride diffusion coefficients after different exposure times to a 3.5% NaCl solution for PC and LC^3^-50 concretes. Based on data from [176].

It has been reported that the diffusion coefficients of chlorides in LC^3^-50 are lowest when compared with other alternative binders, and especially if they are compared with PC (Figure 10) [175,176], with the diffusivity of chlorides in LC^3^-50 being up to 5 to 10 times lower than PC [176,177,178]. The amount of kaolinite in the clay also plays an important role in chloride diffusion. It has been reported that using clays with kaolinite contents of 50% and 80% significantly reduces the critical pore size, thus decreasing the chloride transported through LC^3^-50 mortars [171,179]. The refinement degree of the pore network is achieved much faster in LC^3^-50 than in PC due to the high reactivity of metakaolin, increasing the resistance to chloride ingress at early ages [180]. All these results were obtained without applying current to force chloride transport.

Inside LC^3^-50, as in other cementitious binders, chlorides can be free in the pore solution or immobilized by chemical or physical bounds. The dominant chemical binding mechanism is related to carboaluminates, which react with chloride ions forming Friedel’s salt [171]. It has been reported that more chlorides are chemically bounded in LC^3^-50 than in PC [181]. However, the ability of LC^3^-50 for chemically binding chloride is lower than those of other binders without limestone [182].

From a physical binding perspective, the quantity of chlorides that can be bound to the C-(A)-S-H structure is related to the amount of aluminum available, allowing higher bound content in binders with increased aluminum content [183]. The presence of metakaolin in LC^3^-50 has both negative and positive effects on its physical chloride binding ability [181]. Regardless, the amount of physically bound chlorides in LC^3^-50 is smaller than the amount of chemically bound chlorides [171].

Moreover, as occurs for PC, the decrease in alkalinity of the pore solution greatly influences chloride binding (as discussed for HC in Section 4), when the Friedel’s salt to which chlorides are bound dissolves [184,185]. For this reason, several studies have been investigated the effect of the carbonation of LC^3^-50 on the chloride binding ability [182]. The presence of sulfates also negatively affects the stability of the Friedel’s salt, making the environment especially aggressive when sulfate and chloride are both present [186].

### 5.4. Corrosion of Steel Embedded in LC^3^-50

Other factors such as the alkaline reserve, the morphology of the steel–mortar/concrete interface or the oxygen access can play key roles for the onset and the kinetics of the corrosive attack [132], besides the determining role that the diffusion of the corrosive agents can play in the corrosion kinetics of the steel. Hence, it is advisable to carry out corrosion studies of reinforcing bars embedded in LC^3^-50 concretes before manufacturing reinforced structures. Contributions from the literature published to date are shown in Table 2. In general, the corrosion monitoring studies performed up to now are based on non-destructive electrochemical techniques such as OCP, linear polarization and EIS (Table 2). It is worth mentioning that only one of the studies included in Table 2 mentions the use of superplasticizer [161], although not its amount. As previously said, the use of water-reducing agents in LC^3^-50 formulation is highly important [156], and its absence implies highly porous materials that cannot accomplish the in-service requirements. If this additive is not used for the LC^3^-50 formulation, corrosion results for the steel can be impaired, as aggressive elements easily diffuse through the mortar/concrete.

The passivation ability of steel has been studied in LC^3^-50 pore extracts [192]. Results indicate that steel passive films can form and become stable in LC^3^-50 in the absence of corrosive agents. However, the passive film has less iron oxides than in PC, and may include calcium [192]. Moreover, an external layer of AFm crystals above the passive film, which decrease the corrosion rate in the passive state, has been identified [192]. When 0.5% chlorides are added, the chloride threshold ([Cl^−^]/[OH^−^]) determined for steel is higher in LC^3^-50 pore solution than in PC [192], and this fact is also related to the AFm layer precipitated on the passive layer.

**Figure 11:**
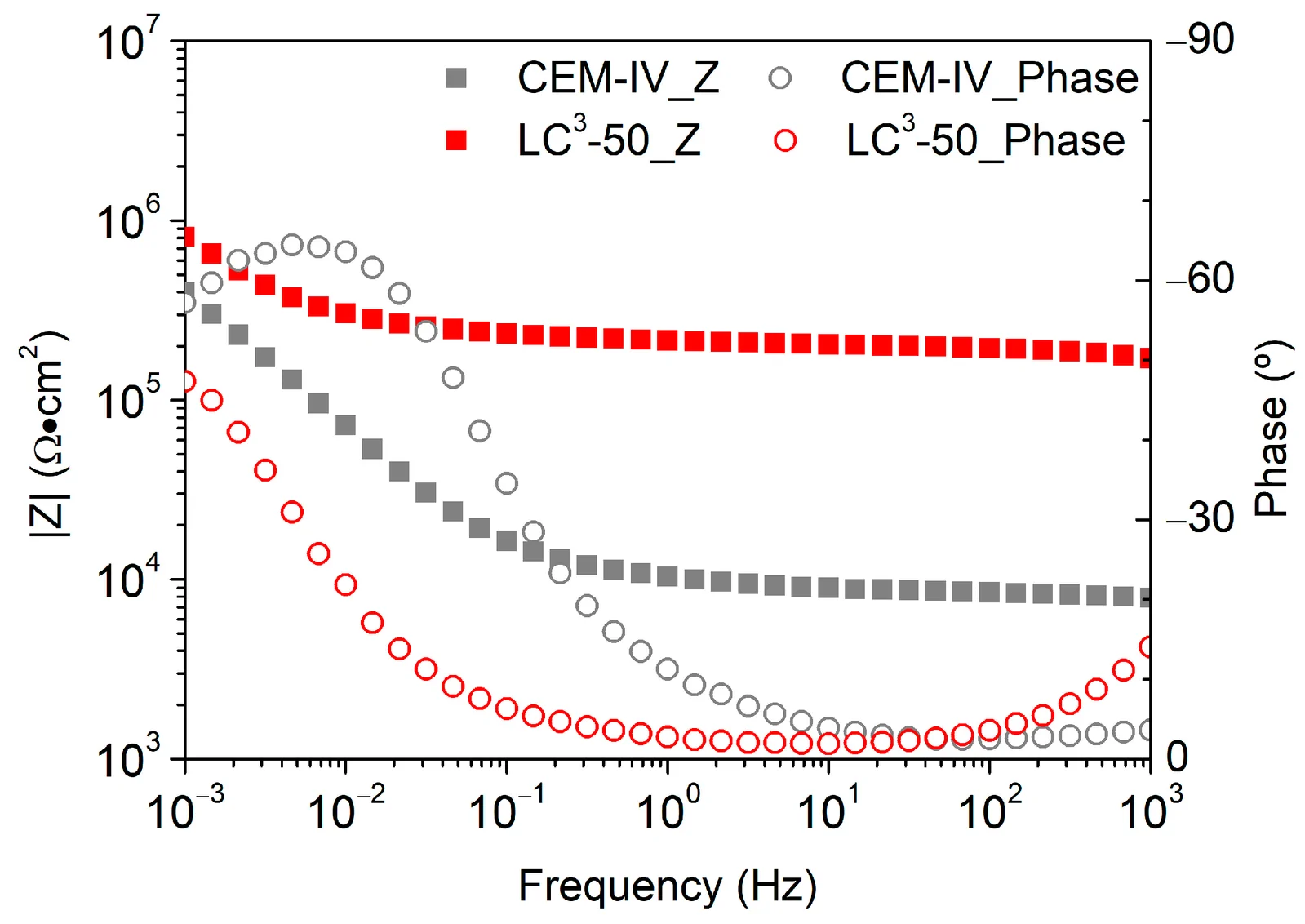
EIS spectra of the steel embedded in CEM IV and LC^3^-50 mortars measured after being partially immersed in 1% NaCl for 140 days. The w/b for CEM IV is 0.5. The LC^3^-50 has a w/b of 0.43, with 0.9% of superplasticizer (polycarboxylate ether). The clay from which the LC^3^-50 was manufactured has 82% kaolinite [194].

In the publications included in Table 2 that resort to EIS measurements, all the studies used 10^−2^ Hz as the lowest frequency [164,187,189,190,191,192]. Although extending the measurements up to lower frequencies makes the measurements a much more time-consuming strategy, it also allows interesting information to be obtained about the electrochemical process taking place on the surface of the bars that is often missed in these shorter spectra. It is easy to obtain reliable information for these systems in measurements carried out up to 5 mHz or even up to 1 mHz in mortars from commercial cements [132] and alternative binders (Figure 7 and Figure 11). This problem becomes especially serious due to the low, highly refined porosity of LC^3^-50 binders—especially when they are manufactured with low water/cement ratios to improve their durability properties—which makes mortars/concretes manufactured from them highly resistive. As has already been discussed, for EIS measurement, resistivity has often proven to be a factor possibly affecting the monitoring techniques. The EIS spectra found in the literature are mostly dominated by the binder resistance and it is not possible to obtain any reliable information [164,187,189,190,191] about the charge transfer resistance of the corrosion process, as can be seen particularly in Figure 11 for steel-reinforced mortars partially immersed in 1% NaCl solution. In these cases, the obtained values after simulation for this key parameter could be considered unreliable. Attempting to use equivalent validated for conductive pore solutions [4,193] or steel exposed in less aggressive conditions [133] can lead to unreliable conclusions.

Bulk resistivities three times higher than that of reference PC concrete have been reported [15] with just 20% clay substitution. This high resistivity can also interfere with the linear polarization measurements [187] if it is not properly compensated. Carrying out this ohmic drop compensation has been attempted using a current interrupt approach for cements with 20% calcined clay substitution [15]. However, other authors, studying typical LC^3^-50 with 50% clinker, have tried to compensate the ohmic drop by current interrupt as well as by positive feedback. However, they report that their outcomes were unsuccessful [187], as they were noisy and hindered an accurate determination of polarization resistance. As the scan rate during polarization resistance measurements can play an important role [195], 0.05 mV/s has been suggested as a sweeping rate for LC^3^-50 [187]. In any case, it is important to stress that other researchers do not indicate if this compensation is taken into account [161,189,190], and there are also authors that, in spite of acknowledging the influence of the ohmic drop [130], do not compensate it.

Alternative methodologies such as the galvanostatic pulse technique have been suggested in the literature to obtain reliable corrosion data in highly resistive materials as it allows to differentiate the influence of the concrete resistance from the resistance of the corrosion process [196,197]. However, no successful attempt to apply this strategy to LC^3^-50 concretes has been reported to date. In any case, it could be assumed that in systems where polarization tests are carried out without ohmic drop compensation [161,189,190], the apparent polarization resistances obtained should be the sum of the real polarization resistance of the steel and the mortar resistance. Hence, the conclusions informing about the worse behavior of the steel in LC^3^-50 than in PC would be the same, but with an increased difference between the corrosion rates.

LC^3^-50 binders are innovative, promising binders of interest because of their low clinker content and not using supplementary cementitious materials with expected reduced availability. Aspects like the nature of clays, the shrinkage, the optimization of different superplasticizers or the standardization have been previously marked as technical criticalities for LC^3^-50 development [198]. However, their potential use in reinforced structures requires a deeper analysis as their lower alkaline reserve can compromise the corrosion resistance of the embedded steel under aggressive conditions. Selecting clays with a high kaolinite content for manufacturing the LC^3^-50 can be a key point for obtaining proper steel durabilities in corrosion tests.

**Figure 12:**
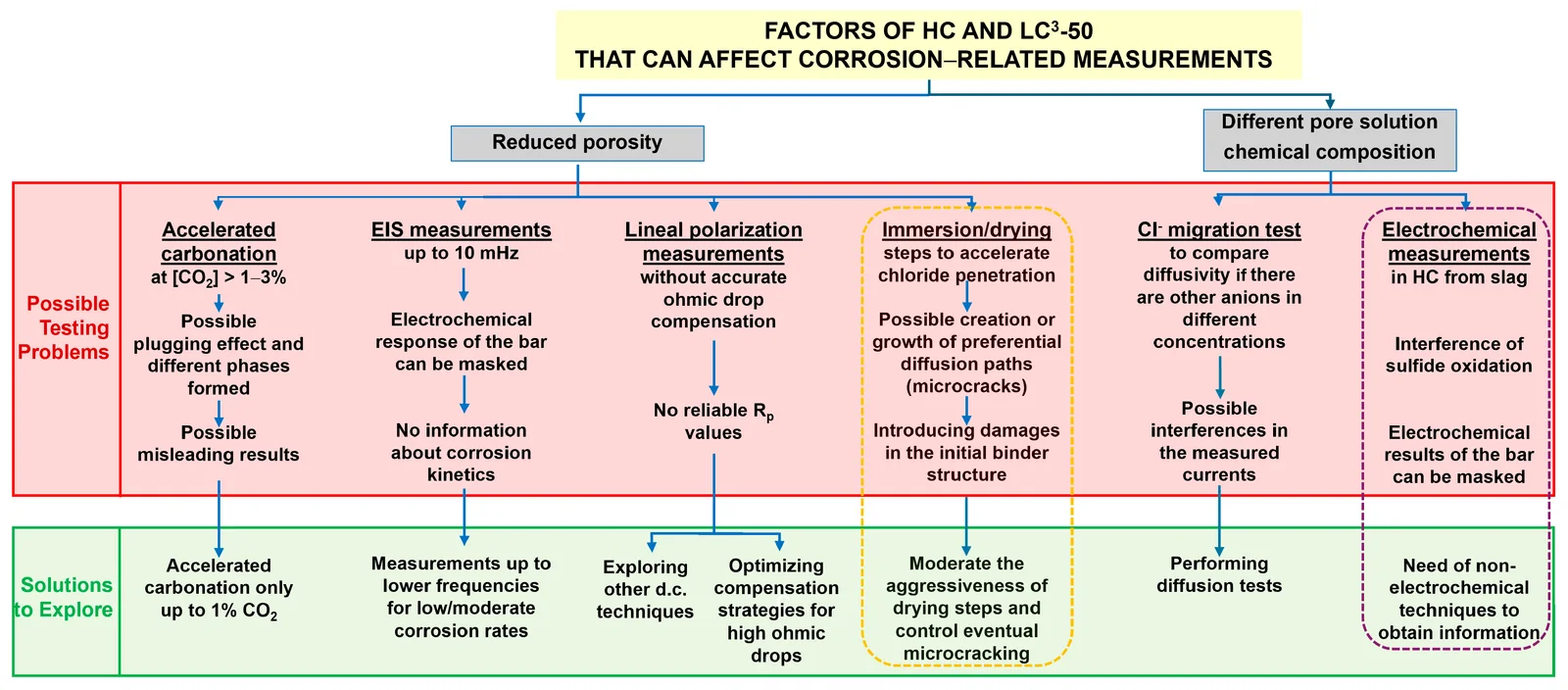
Scheme including factors of HC and LC^3^-50 binders that can affect corrosion monitoring of embedded steel reinforcements. The violet dashed line indicates a problem that does not appear in LC^3^. The orange dashed line problem has not been identified LC^3^-50 systems.

The technical problems identified in studies dealing with corrosion-related aspects of steel embedded in HC and LC^3^-50 are summarized in Figure 12. The reasons why the experimental methodology valid for PC can be misleading are included, as well as some possible strategies to overcome these issues. All the problems cited in Figure 12 have been identified for HC, while the one surrounded by a violet dashed line does not appear for LC^3^ concretes because of their compositions, and the one surrounded by an orange dashed line has not been identified LC^3^-50 systems.

## 6. Conclusions and Remarks

HC and LC^3^-50 binders are environmentally friendly alternatives to the clinker-rich commercial cements that do not have the technical limitations for in-construction use of widely explored AAMs. However, if these still less explored innovative binders are considered for use in reinforcing steel structures in the future, the following points about material formulations are worth bearing in mind:

1. The refined porosity of many AAM and HC binders, which can be so beneficial for the mechanical properties, favors the generation of microcracks and preferential diffusion paths for corrosive agents and compromises the durability of embedded steel. Hence, very intensive activation of AAMs or HCs can be counterproductive for structures with embedded steel. The use of solid salts without highly caustic character for activation of HC is a strategy that contributes to making an eventual implementation feasible. The activators considered for HC when steel is going to be embedded must be salts not only able to avoid microcracking generation, but that do not release corrosive ions into the pore solutions.
2. The low alkaline reserve of LC^3^-50 has proven to be able to negatively affect the performance of steel when the system is exposed to carbonation or chloride tests. Selecting clays with a high kaolinite content for manufacturing the LC^3^-50 and adding proper amounts of superplasticizers could be key points for obtaining steel durabilities in corrosive environments closer to those of commercial binders. The effectiveness of corrosion protection that the AFm precipitated phase can offer to the steel in pore solutions must be verified in mortar or concrete tests.

Regarding the present evidence, it is important to consider the following points regarding the use of traditional testing methods designed for PC when alternative binders are characterized:

1. Resorting to high CO_2_ concentrations in accelerated carbonation tests especially distorts the mechanism of the process in alternative binders whose porosity is much more refined than that of PC.
2. As the use of migration tests to obtain rapid information about the chloride transport in AAMs has been considered unreliable due to the differences existing between the composition of pore solution, microstructure and chloride binding mechanism in this binder and in PC, the reliability for the use of this test in HC and LC^3^-50 needs to be confirmed.
3. If HCs are manufactured from slag and the corrosion of the embedded steel is intended to be electrochemically monitored, the interference of the sulfide oxidation process in the results must be considered.
4. When highly resistive concretes or mortars are considered and the embedded steel corrodes at a meaningful rate, it is difficult to obtain reliable information about the corrosion kinetics by EIS, as most of the spectrum is dominated by the resistance of the binder. This problem must be always borne in mind if low-porous HC or LC^3^-50 are considered.
5. If the polarization resistance is intended to be measured for corroding steel embedded in highly resistive mortars or concretes such as well-formulated LC^3^-50, the ohmic drop through the cover must be properly compensated to obtain reliable information about the electrochemical process taking place on the reinforcements. Optimizing methods for reliable ohmic drop compensation that allows easy corrosion rate measurements with d.c. techniques could be considered a clear priority research.
6. For HC binders using slag as precursor, mass loss evaluation, section loss, or pit depth must be considered in the experimental design, as electrochemical measurements become unreliable. More data on these non-electrochemical techniques would be necessary in the current state of the art.

## Figures and Tables

**Table 2:** Corrosion studies of steel embedded in LC^3^-50 binders.

Article	Material	Kaolinite in Clay	Corrosive Conditions	Corrosion Tests Reported	Conclusions About Steel in LC^3^-50
[187]	Mortar w/b: 0.5	Not reported	Wetting/drying cycles in NaCl solution for 70 days	Linear polarization EIS Visual observation	Polarization resistance without ohmic drop larger for LC^3^-50 than for PC
[188]	Mortar w/b: 0.5 and 0.6	Not reported	Carbonated at 4% CO_2_ and 65% RH	Linear polarization	Increasing w/c ratios implies increased corrosion rate
[189]	Mortar w/b: 0.4	Not reported	Wetting/drying cycles in NaCl solution (up to 18 cycles)	OCP Linear polarization EIS	Propose method for chloride threshold in mortar
[190]	Paste w/b: 0.45	Not reported	Added chloride (0.18%) in manufacturing + carbonation at 20% CO_2_, 70% RH for 14 days	OCP Linear polarization EIS	Depassivation in LC^3^-50 is faster than in PC
[191]	Concrete w/b: 0.38	43%	Buried in simulated aggressive soil + exposed to 16 wetting/drying cycles in saline solution	EIS	LC^3^-50 shows more reduced corrosion rate than PC
[166]	Mortar w/b: 0.485	70%	20% CO_2_, 80% RH at 30 °C for 28 days + 13 wetting/drying cycles NaCl solution	OCP Linear polarization Cyclic polarization	Corrosion activity in LC^3^-50 is higher than that of PC
[161]	Concrete w/b: 0.50	40%	Carbonated at 3% CO_2_, 20 °C and 60% RH for 112 days	OCP Linear polarization EIS	Steel more vulnerable to corrosion in LC^3^-50 than in PC
[192]	Pore solution	Not reported	As-cured and with added chlorides	OCP Linear polarization EIS	Carbon steel in the LC^3^-50 pore solution has a higher chloride threshold than in PC
[164]	Mortar w/b: 0.50	Not reported	Carbonated at 3% CO_2_, and 65% RH + partial immersion cycles in water for 420 days	OCP Linear polarization EIS	Increased corrosion rate when exposed to higher RH

## Data Availability

No new data were created or analyzed in this study. Data sharing is not applicable to this article.
